# Cardiovascular changes in patients with adult-onset growth hormone deficiency assessed by CMR

**DOI:** 10.1186/1532-429X-14-S1-P192

**Published:** 2012-02-01

**Authors:** Abhishek Dattani, Julia Thomas, Filip Zemrak, Thomas R Burchell, Steffen E Petersen, Ashley Grossman, Marta Korbonits, Ceri Davies

**Affiliations:** 1 Cardiovascular Biomedical Research Unit, Barts and the London School of Medicine and Dentistry, Queen Mary, University of London, London, UK; 2 Centre for Endocrinology, William Harvey Research Institute, Barts and the London School of Medicine and Dentistry, Queen Mary, University of London, London, UK; 3 Oxford Centre for Diabetes, Endocrinology and Metabolism, University of Oxford, Oxford, UK

## Summary

The study investigated cardiovascular changes in adult-onset growth hormone deficiency (GHD) and showed that patients with adult-onset GHD have a left ventricular mass index (LVMi) at or below the lower limit of normal, which improves with one year of growth hormone replacement.

## Background

GHD causes cardiovascular problems, with loss of cardiac response to exercise and increased cardiac mortality, however the underlying processes are poorly understood.

## Methods

Ten patients with adult-onset GHD and age- and sex-matched controls (n=10) underwent CMR. Patients underwent scans before disease treatment and at twelve months after treatment. Cardiac parameters were calculated and indexed to body surface area (BSA). The comparison between groups was done using Mann-U-Whitney test and within the group using Wilcoxon test. The data are presented as median values.

## Results

Patients with GHD did not have significantly different left ventricle (LV) mass or volumetric parameters from controls: LV mass index (LVMi) 55.0 vs. 56.6 g/m^2^, p=0.315; end diastolic volume index (EDVi) 69.2 vs. 76.3 ml/m^2^, p=0.1655; end systolic volume index (ESVi) 24.8 vs. 28.3 ml/m^2^, p=0.3527 and ejection fraction (EF) 64 vs. 63 %, p=0.8197. However, patients had an LVMi beneath the lower limit of normal when compared to published normal ranges (55.4 - 74.0 g/m^2^).

There were no differences between right ventricular (RV) EDVi (68.0 vs. 80.8 ml/m^2^, p=0.393), ESVi (26.2 vs. 27.5, p=0.7394) and EF (63 vs. 61 %, p=0.9696) in the GHD group and controls.

Patients with GHD on GH treatment for 1 year showed an increase in median insulin-like growth factor I (IGF-I) SDS from -1.83 to +0.40 (p=0.0068). There was no correlation between LVMi and IGF-I SDS (r=0.164, p=0.657). At one year the median LVMi moved into the previously published normal range (55.0 to 63.0 g/m^2^, normal range 55.4 - 74.0) achieving statistical significance compared to pre-treatment values (p=0.0156).

## Conclusions

Patients with adult-onset GHD have an LVMi at or below the lower limit of normal, which improves with one year of growth hormone replacement.

## Funding

Supported by departmental grants by Pfizer and Novartis.

